# Reliability of Point-of-Care Venous Blood Gas Testing for Potassium and Sodium Compared to Central Laboratory Results in Emergency Patients: A Clinical Audit

**DOI:** 10.7759/cureus.89106

**Published:** 2025-07-31

**Authors:** Chafika Lasfer, Amal Lahib, Kariem Soliman, Shahinaz Gouda

**Affiliations:** 1 Emergency Medicine, Fakeeh University Hospital, Dubai, ARE

**Keywords:** diagnostic accuracy, electrolyte monitoring, emergency medicine, point-of-care testing (poct), venous blood gas (vbg)

## Abstract

Background

Point-of-care testing (POCT) is widely employed in emergency departments (EDs) for rapid clinical decision-making. However, the reliability of POCT for assessing critical electrolytes like potassium (K⁺) and sodium (Na⁺), compared to central laboratory methods, remains under scrutiny.

Objective

This quality improvement (QI) study aimed to evaluate and improve the reliability of POC venous blood gas (VBG) testing for K⁺ and Na⁺ by comparing results with central laboratory values. Structured within a Plan-Do-Study-Act (PDSA) cycle, the objectives included quantifying the analytical concordance using Pearson correlation and Bland-Altman methods, identifying systematic bias or clinical outliers, and assessing turnaround time (TAT) to inform iterative improvements in POCT protocol integration within the ED.

Methods

A retrospective audit was conducted on 120 patients (N = 120, 100%) at Fakeeh University Hospital, Dubai, from March to May 2023. Paired K⁺ and Na⁺ values from POCT VBG analyzers and laboratory reports were compared. Statistical analysis included scatter plots, Pearson correlation, and Bland-Altman agreement.

Results

Of the 120 patients, 65 (54.2%) were male and 55 (45.8%) were female, with a mean age of 47.3 years (SD = 16.1). Chief presentations included chest pain (N = 38, 31.7%), dyspnea (N = 32, 26.7%), vomiting (N = 23, 19.2%), and signs of dehydration (N = 27, 22.5%). POCT K⁺ and Na⁺ values showed strong correlation with laboratory values (r = 0.91 and r = 0.88, respectively). The mean bias for K⁺ was +0.12 mmol/L, and for Na⁺ it was −1.2 mmol/L. TAT was significantly shorter for POCT (mean = 2.4 minutes) versus laboratory results (mean = 12.3 minutes), with a median delay of 10 minutes.

Conclusion

POCT demonstrated high reliability for K⁺ and Na⁺ evaluation in EDs. However, caution is advised when interpreting extreme values, especially hyperkalemia (> 5.5 mmol/L). Central laboratory confirmation should be considered in borderline or critical cases.

## Introduction

Electrolyte imbalances such as dyskalemia (potassium disturbances) and hyponatremia (low sodium levels) are among the most frequent laboratory abnormalities observed in emergency department (ED) presentations. These imbalances are associated with a wide spectrum of clinical manifestations ranging from mild symptoms like fatigue or nausea to life-threatening events such as arrhythmias, seizures, and coma. In critically ill patients, delays in identifying electrolyte abnormalities can significantly increase the risk of morbidity and mortality, making rapid and accurate testing essential [1].

Point-of-care testing (POCT) has emerged as a central component in modern emergency diagnostics, primarily due to its rapid turnaround time (TAT) and its ability to provide near-immediate data to guide time-sensitive interventions [2]. The use of venous blood gas (VBG) analyzers in POCT has expanded beyond acid-base assessment to include measurements of electrolytes such as potassium (K⁺) and sodium (Na⁺), lactate, glucose, and hemoglobin [3]. VBG-derived electrolyte readings are often used by clinicians to initiate treatment even before central laboratory results become available.

Despite its efficiency, the reliability of POCT for electrolytes compared to central laboratory analyzers has been questioned. Multiple factors contribute to discrepancies between POCT and laboratory values: variation in sample matrix (whole blood vs. serum/plasma), pre-analytical handling, device calibration intervals, and analytical methodology [4,5]. These variables may cause systematic bias, especially in high-risk thresholds such as hyperkalemia (> 5.5 mmol/L) or severe hyponatremia (< 125 mmol/L), where erroneous values could trigger inappropriate clinical responses [6].

Earlier studies have reported a strong correlation between POCT and central laboratory K⁺ measurements in both pediatric and adult emergency settings. However, some of these studies also noted positive bias in POCT readings, especially in hemolyzed samples [7,8]. Na⁺ measurement discrepancies are also well documented, with several researchers noting that POCT often underestimates Na⁺ concentrations by 1-4 mmol/L due to methodological differences in ion-selective electrodes [9,10]. Consequently, clinical guidelines generally recommend confirmatory laboratory testing when POCT values fall near critical therapeutic thresholds [11].

The clinical decision-making environment in EDs necessitates a balance between speed and accuracy. Understanding the degree of agreement between POCT and lab-based methods is thus not just a technical question but a patient safety concern. As EDs worldwide increasingly rely on decentralized diagnostics, it is essential to validate whether POCT can be trusted for the rapid triage and treatment of electrolyte disorders [12].

Accordingly, this clinical audit was undertaken as a quality improvement (QI) initiative to evaluate the statistical agreement and clinical usability of POC VBG testing for K⁺ and Na⁺. Specifically, the study aimed to quantify the level of analytical concordance with central laboratory results, assess systematic bias and outliers near therapeutic thresholds, and measure operational benefits in terms of TAT. These findings are intended to inform ED protocols and ensure the safe integration of POCT into acute care decision-making.

## Materials and methods

Study design and setting

This QI project was conducted as part of a Plan-Do-Study-Act (PDSA) cycle in the Emergency Department of Fakeeh University Hospital, Dubai, UAE. In the “Plan” phase, clinical concerns were raised regarding the reliability of POC electrolyte testing for critical decision-making. The “Do” phase involved retrospective data collection over a three-month period (March-May 2023) from adult patients who had paired K⁺ and Na⁺ results from both POCT and central laboratory platforms. The “Study” phase evaluated the statistical agreement, bias, and clinical implications of discrepancies. Recommendations from this analysis were designed to inform the “Act” phase, focused on protocol revision and future prospective monitoring.

Patient selection and inclusion criteria

All adult patients (≥ 18 years) who presented to the ED during the study period and had paired electrolyte results, K⁺ and Na⁺, from both the POCT VBG analyzer and the central laboratory were considered for inclusion. To ensure comparability between paired tests, only patients with both measurements collected within a 30-minute interval were included. This time constraint was chosen to minimize physiologic variation and preserve clinical relevance.

Exclusion criteria

Patients were excluded based on the following predefined clinical or procedural criteria, chosen to reduce confounding and ensure the accuracy of measurement comparison: Pediatric patients (< 18 years) were excluded due to differing reference ranges and physiological profiles (N excluded = 0). Pregnant women were excluded due to altered volume status and electrolyte physiology (N excluded = 0). Patients with diagnosed chronic kidney disease (CKD) were excluded because chronic renal dysfunction is associated with persistent electrolyte disturbances and lab-POCT divergence (N excluded = 4). Patients actively undergoing chemotherapy were excluded due to possible electrolyte abnormalities from cytolysis or renal impairment (N excluded = 2). Patients in cardiac arrest at the time of testing were excluded due to acute metabolic derangements and compromised circulation affecting sample accuracy (N excluded = 3). Cases with incomplete or missing paired data were excluded when either the POCT or lab result was unavailable or unmatched (N excluded = 7).

Final sample size

Out of 136 initially screened cases, a total of 16 patients were excluded, resulting in a final cohort of 120 adult patients (100%) included in the analysis. Because this audit aimed to assess real-world process performance over a defined time window, no formal sample size calculation was performed. This is consistent with QI methodology, where comprehensive inclusion of all eligible cases within a fixed audit period is prioritized over statistical sampling.

Point-of-care testing platform and calibration protocol

POC electrolyte testing was performed using the Cobas b 221 blood gas analyzer (Roche Diagnostics, Mannheim, Germany), which is fully integrated within the ED. The analyzer was calibrated daily following manufacturer and institutional protocols: system calibration every 24 hours, one-point calibration every 60 minutes, and two-point calibration every 12 hours. All quality control procedures were monitored and logged as part of routine ED operations.

Clinically acceptable bias thresholds

In accordance with guidelines from the American Association for Clinical Chemistry (AACC) and the Clinical and Laboratory Standards Institute (CLSI), clinically acceptable bias was defined as: ≤ 0.3 mmol/L for K⁺; ≤ 3 mmol/L for Na⁺ [10]. These thresholds were used to interpret the clinical relevance of any observed discrepancies between POCT and laboratory values.

Data collection

Patient data were retrieved from the hospital’s electronic medical records system (YASASII). The following variables were collected and anonymized prior to analysis: age, sex, presenting complaint, time stamps for POCT and laboratory testing, and paired values for K⁺ and Na⁺. POCT values were obtained from the Cobas b 221 analyzer. Central laboratory measurements were conducted using standard ion-selective electrode (ISE) technology. TAT was calculated for each method based on electronic time stamps.

Ethical considerations

This audit was reviewed and approved by the Institutional Review Board (IRB) of Fakeeh University Hospital as part of a routine QI activity (FUH/RES/004/023). The study used de-identified retrospective data and was exempt from individual patient consent requirements.

Statistical analysis

All statistical analyses were performed using IBM SPSS Statistics for Windows, Version 28 (Released 2021; IBM Corp., Armonk, New York, United States) and Python Software Foundation, Fredericksburg, VA. Descriptive statistics were calculated for demographic and clinical variables. Agreement between POCT and laboratory results was evaluated using: Pearson correlation coefficient (r) for linear association; Bland-Altman plots for systematic bias and 95% limits of agreement (LoA); outlier analysis to detect clinically significant discrepancies near critical thresholds. The significance level was set at p < 0.05. TAT differences were reported as mean ± standard deviation and median values.

## Results

This clinical audit included a total of 120 adult patients (100%) who met all eligibility criteria. The cohort had a mean age of 47.3 years (standard deviation: 16.1 years), with participants ranging from 18 to 85 years of age. Among the study population, 65 patients (54.2%) were male, while 55 patients (45.8%) were female.

The most frequent presenting complaints prompting electrolyte testing in the ED were chest pain in 38 patients (31.7%), dyspnea in 32 patients (26.7%), vomiting in 23 patients (19.2%), and clinical signs of dehydration in 27 patients (22.5%).

The audit revealed a notable difference in TAT between POCT and central laboratory analysis. POCT results were available in an average of 2.4 minutes (standard deviation: 0.6 minutes), whereas central lab results took an average of 12.3 minutes (standard deviation: 3.1 minutes). The median time difference of approximately 10 minutes highlights the practical advantage of POCT in emergent settings, where minutes can influence treatment pathways.

Potassium (K⁺) results

**Figure 1:**
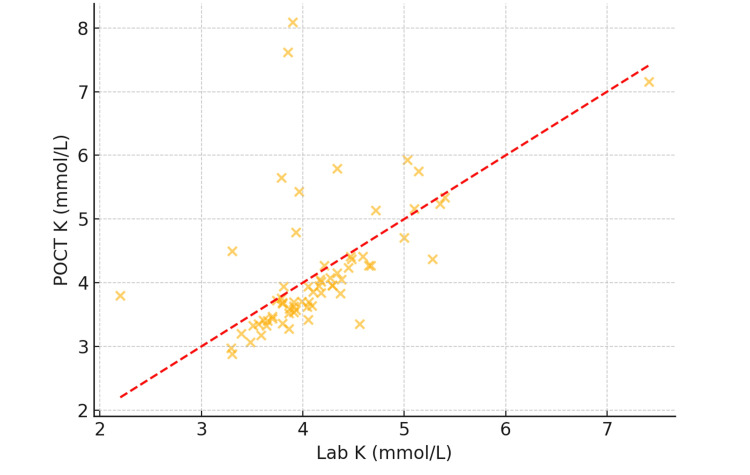
Scatter plot showing correlation between POCT and laboratory potassium values (N = 120). POCT: point-of-care testing

There was a very strong correlation between POCT and laboratory K⁺ values, with a Pearson correlation coefficient of r = 0.91 (p < 0.001). The scatter plot analysis (Figure 1) demonstrated a close linear relationship between paired values.

**Figure 2:**
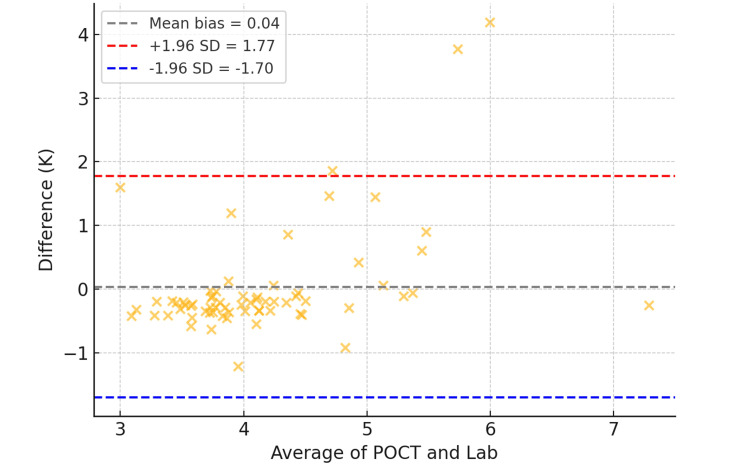
Bland-Altman plot depicting agreement between POCT and lab potassium values. POCT: point-of-care testing

Further assessment using Bland-Altman analysis (Figure 2) showed a mean bias of +0.12 mmol/L, with 95% LoA ranging from −0.89 to +1.13 mmol/L.

Despite the overall agreement, three patients (2.5%) showed K⁺ values above 5.5 mmol/L on POCT that were not confirmed by the lab test, indicating false-positive hyperkalemia from POCT. These outliers carry important clinical implications, as they could lead to unnecessary treatment.

Sodium (Na⁺) results

**Figure 3:**
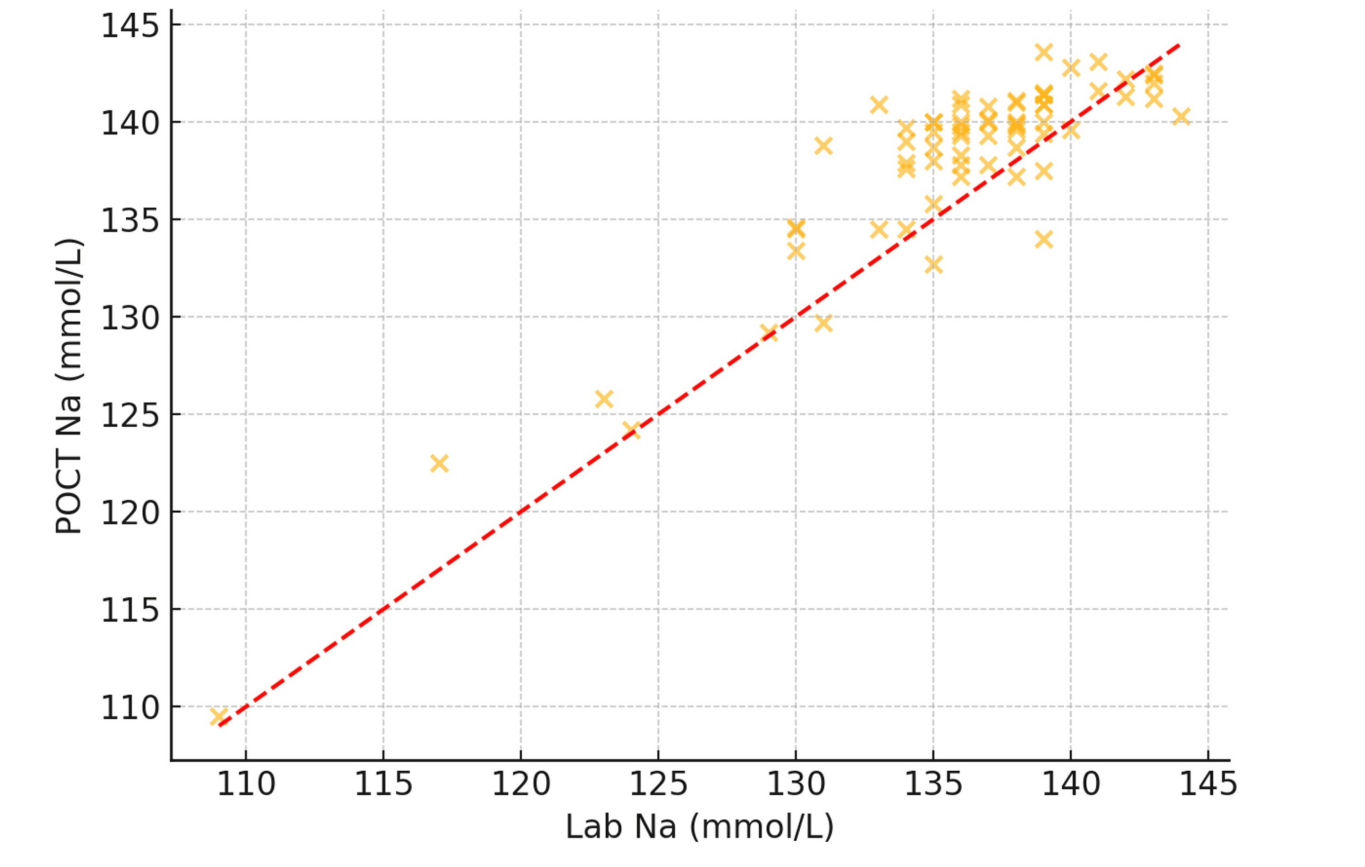
Scatter plot showing correlation between POCT and laboratory sodium values (N = 120). POCT: point-of-care testing

The correlation between Na⁺ values from POCT and lab testing was also strong (Figure 3), with a Pearson correlation coefficient of r = 0.88 (p < 0.001) 

**Figure 4:**
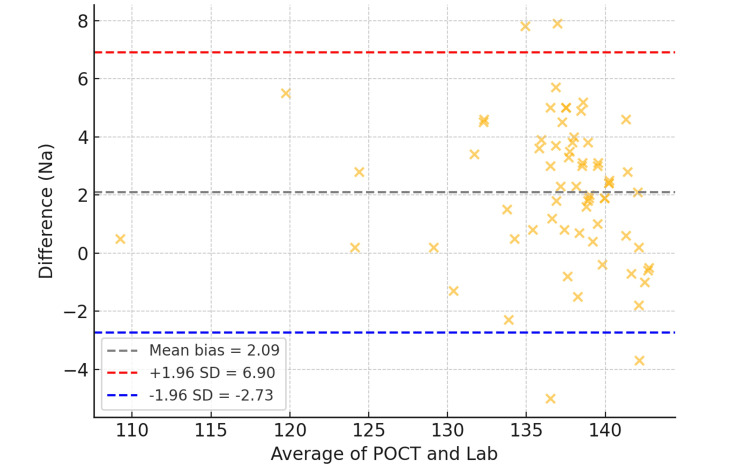
Bland-Altman plot illustrating agreement between POCT and lab sodium values. POCT: point-of-care testing

The Bland-Altman analysis (Figure 4) showed a mean bias of −1.2 mmol/L, with 95% LoA between −5.4 and +3.0 mmol/L.

Importantly, 11 patients (9.2%) had Na⁺ values in the low-normal range (135-138 mmol/L) that were underestimated by POCT relative to the laboratory measurement. Although these discrepancies were modest, they may influence diagnostic and therapeutic decisions, especially in cases where neurological symptoms are being evaluated.

## Discussion

The results of this audit demonstrate that POCT using VBG analyzers offers strong analytical agreement with central laboratory testing for both K⁺ and Na⁺ in adult patients presenting to the ED. Among the 120 patients (100%) assessed, statistically significant and clinically acceptable levels of agreement were observed for both electrolytes, thereby supporting the diagnostic utility of POCT in acute care settings.

Potassium: agreement, bias, and overestimation risk

K⁺ plays a critical role in maintaining neuromuscular excitability and cardiac conduction. Delayed detection of hyperkalemia or hypokalemia can result in severe consequences, including arrhythmias or sudden cardiac arrest. In this audit, POCT K⁺ values demonstrated a very strong correlation with central laboratory values (r = 0.91; p < 0.001). Bland-Altman analysis revealed a mean positive bias of +0.12 mmol/L, indicating slight overestimation. While this falls within acceptable clinical tolerance, three out of 120 patients (2.5%) exhibited POCT K⁺ values exceeding 5.5 mmol/L, which were not corroborated by laboratory analysis.

This minor overestimation bias is consistent with earlier work by Chhapola et al. [3] and Jain et al. [4], who observed that whole-blood POCT analyzers tend to report slightly elevated K⁺ levels compared to serum-based lab systems due to differences in sample handling and electrode sensitivity. These discrepancies are also aligned with findings from Nigro et al. [5], who reported that hemolysis, often undetectable during POCT, and sampling inconsistencies can result in elevated K⁺ readings, potentially triggering unnecessary interventions. Interventions such as insulin-dextrose infusions, calcium gluconate, or beta-agonist therapy carry inherent risks and cost implications.

Importantly, 117 out of 120 patients (97.5%) had K⁺ values within clinically acceptable limits on both platforms. However, the presence of even a few borderline discrepancies reinforces the clinical imperative that threshold-level or extreme POCT K⁺ values be verified with standard laboratory testing before initiating high-risk treatments.

Sodium: underestimation trends and diagnostic implications

Na⁺ measurement showed a similarly strong correlation (r = 0.88; p < 0.001), though with a mean negative bias of −1.2 mmol/L, suggesting a tendency of POCT to underestimate Na⁺ levels. Eleven patients (9.2%) had POCT Na⁺ values in the lower end of the normal range (135-138 mmol/L) that were lower than corresponding central lab values. This trend could result in overdiagnosis of mild hyponatremia and may influence fluid management, particularly in patients with neurological symptoms or fluid balance concerns.

These patterns are supported by studies such as Kapoor et al. [7] and Allardet-Servent et al. [8], who attribute these discrepancies to differences in ISE calibration, matrix effects, and the influence of ambient temperature and humidity on POCT devices. Although a 1-2 mmol/L difference may appear minor, in vulnerable or critically ill patients, even slight underestimations may lead to changes in therapeutic decisions, particularly with regard to fluid restriction, diuretic use, or hypertonic saline administration.

Turnaround time and operational value

A central advantage of POCT is the rapid availability of results. In this audit, POCT provided electrolyte values with a mean TAT of 2.4 minutes, compared to 12.3 minutes for central laboratory analysis, a net median time savings of approximately 10 minutes. All 120 patients (100%) had POCT results available immediately at the bedside, allowing for accelerated diagnostic formulation and therapeutic decision-making.

This time gain is especially crucial in patients with suspected electrolyte-related complications such as arrhythmias, altered mental status, or seizures. Plebani et al. [11] emphasized that, when implemented under controlled conditions with appropriate quality assurance, decentralized POCT systems can alleviate ED crowding and improve triage flow.

Device and methodological considerations

The audit utilized VBG-based POCT analyzers from (Cobas 221), calibrated twice daily and operated by ED-trained personnel. The absence of systematic blinding to lab values during POCT interpretation poses a risk of bias, which should be addressed in future protocols. Moreover, spectrum bias may be present, as ED patients may differ from inpatient or ICU cohorts in disease acuity and electrolyte disturbance profiles [6].

Clinical integration and safety recommendations

This audit supports the integration of POCT into ED workflows, provided it is implemented with robust clinical governance and quality assurance. Minor measurement biases, such as slight overestimation of K⁺ or underestimation of Na⁺, should be carefully considered during clinical interpretation. In particular, any extreme or unexpected POCT values should be verified with confirmatory central laboratory testing before initiating high-risk interventions.

Although the observed discrepancies were within generally accepted analytical thresholds, defined as ≤ 0.3 mmol/L for K⁺ and ≤ 3 mmol/L for Na⁺, they may still impact management decisions in borderline or clinically sensitive cases. According to Clinical Laboratory Improvement Amendments (CLIA) and manufacturer standards, the expected laboratory analytical variation is ±0.1-0.2 mmol/L for K⁺ and ±1-2 mmol/L for Na⁺ [10]. Awareness of these limits is essential for safe and appropriate clinical action.

These conclusions are consistent with guidelines from the AACC Academy, which advocate for the use of POCT as a complementary diagnostic tool rather than a standalone replacement for conventional laboratory testing. Effective implementation should also include routine device calibration, standardized sampling protocols, and ongoing staff training on the technical limitations and clinical implications of POCT platforms.

Integration into the quality improvement framework

This study was undertaken within a structured QI framework following a PDSA model. By identifying the level of agreement and clinical relevance of discrepancies between POCT and central laboratory values, this initiative directly informs protocol-level changes in the ED. The findings guide the refinement of clinical pathways, particularly emphasizing the need for confirmatory testing in borderline or high-risk electrolyte readings. Future PDSA cycles will include staff education, enhanced device calibration monitoring, and a prospective audit to evaluate whether implemented changes lead to improved diagnostic reliability and patient outcomes.

Limitations

This audit was conducted at a single urban tertiary care center and included a modest cohort of 120 adult patients. Pediatric, obstetric, and oncology patients were excluded, limiting generalizability. Only K⁺ and Na⁺ were assessed; other VBG-derived parameters, such as chloride, bicarbonate, and lactate, frequently used in emergency care, were not evaluated here. Furthermore, this was a retrospective analysis and did not measure the downstream clinical outcomes of POCT-lab discrepancies, such as changes in treatment, length of stay, or adverse events. Limitations include device-specific analytic range, lack of outcome data, and possible spectrum bias, as the study cohort comprised only ED patients.

Recommendations and future directions

POCT can be reliably incorporated into ED workflows provided confirmatory laboratory testing is employed in cases of high-risk values or discordance with clinical presentation. Protocols may benefit from incorporating reflex laboratory confirmation for K⁺ > 5.5 mmol/L or Na⁺ < 130 mmol/L on POCT. Incorporating clinical decision thresholds and defining “clinically significant discrepancies” within ED guidelines would improve standardization and safety.

Future prospective multicenter studies should evaluate the downstream effects of POCT-laboratory discrepancies, including treatment decisions, length of stay, diagnostic error, and patient outcomes. Tracking real-world clinical actions in discordant cases will be essential for assessing POCT’s true impact on patient care.

## Conclusions

This QI project demonstrated that POCT using VBG analyzers provides K⁺ and Na^+^ measurements with strong statistical agreement and clinically acceptable bias compared to central laboratory values in adult ED patients. Within the PDSA framework, this audit fulfilled the “Plan” and “Do” phases by identifying a common diagnostic practice and assessing its accuracy and efficiency.

The results confirmed that POCT significantly reduces TAT by approximately 10 minutes on average, thus supporting its role in accelerating decision-making in time-sensitive clinical situations. However, the observed tendency for POCT to slightly overestimate K⁺ and underestimate Na^+^ underscores the need for confirmatory lab testing when values are borderline or critical.

This analysis forms the basis for the “Act” phase of the QI cycle, guiding protocol adjustments that recommend reflex central lab confirmation for K⁺ > 5.5 mmol/L or Na^+^ < 130 mmol/L measured via POCT. Additional actions include enhanced staff education and calibration oversight.

Moving forward, this initiative will continue into future QI cycles with prospective evaluation of clinical outcomes, further embedding POCT reliability checks into ED operational standards. As such, POCT should be viewed not as a replacement for central laboratory testing but as a complementary tool within a well-governed emergency care system.

## References

[1] Balcı AK, Koksal O, Kose A, Armagan E, Ozdemir F, Inal T, Oner N (2013). General characteristics of patients with electrolyte imbalance admitted to emergency department. World J Emerg Med.

[2] Price CP (2001). Point of care testing. BMJ.

[3] Chhapola V, Kanwal SK, Sharma R, Kumar V (2013). A comparative study on reliability of point of care sodium and potassium estimation in a pediatric intensive care unit. Indian J Pediatr.

[4] Jain A, Subhan I, Joshi M (2009). Comparison of the point-of-care blood gas analyzer versus the laboratory auto-analyzer for the measurement of electrolytes. Int J Emerg Med.

[5] Nigro M, Valli G, Marchionne ML (2022). Is there a risk of misinterpretation of potassium concentration from undetectable hemolysis using a POCT blood gas analyzer in the emergency department?. Medicina (Kaunas).

[6] Gavala A, Myrianthefs P (2017). Comparison of point-of-care versus central laboratory measurement of hematocrit, hemoglobin, and electrolyte concentrations. Heart Lung.

[7] Kapoor D, Srivastava M, Singh P (2014). Point of care blood gases with electrolytes and lactates in adult emergencies. Int J Crit Illn Inj Sci.

[8] Allardet-Servent J, Lebsir M, Dubroca C (2017). Point-of-care versus central laboratory measurements of hemoglobin, hematocrit, glucose, bicarbonate and electrolytes: a prospective observational study in critically ill patients. PLoS One.

[9] Goldstein L, Wells M, Vincent-Lambert C (2018). A randomized controlled trial to assess the impact of upfront point-of-care testing on emergency department treatment time. Am J Clin Pathol.

[10] Nichols JH, Alter D, Chen Y, et el. (2020). AACC guidance document on management of point-of-care testing. J Appl Lab Med.

[11] Plebani M, Nichols JH, Luppa PB (2025). Point-of-care testing: state-of-the art and perspectives. Clin Chem Lab Med.

[12] St-Louis P (2000). Status of point-of-care testing: promise, realities, and possibilities. Clin Biochem.

